# Tics are caused by alterations in prefrontal areas, thalamus and putamen, while changes in the cingulate gyrus reflect secondary compensatory mechanisms

**DOI:** 10.1186/1471-2202-15-6

**Published:** 2014-01-07

**Authors:** Kirsten R Müller-Vahl, Julian Grosskreutz, Tino Prell, Jörn Kaufmann, Nils Bodammer, Thomas Peschel

**Affiliations:** 1 Clinic of Psychiatry, Socialpsychiatry and Psychotherapy, Hannover Medical School, Carl-Neuberg-Street 1, D-30625 Hannover, Germany; 2 Department of Neurology, University Hospital Jena, Jena, Germany; 3 Department of Neurology, Otto-von-Guericke-University, Magdeburg, Germany; 4 Max Planck Institute for Human Development, Lentzeallee 94, Berlin 14195, Germany

**Keywords:** Tic, Tourette syndrome, DTI, Putamen, Thalamus, Cingulate gyrus

## Abstract

**Background:**

Despite strong evidence that the pathophysiology of Tourette syndrome (TS) involves structural and functional disturbances of the basal ganglia and cortical frontal areas, findings from in vivo imaging studies have provided conflicting results. In this study we used whole brain diffusion tensor imaging (DTI) to investigate the microstructural integrity of white matter pathways and brain tissue in 19 unmedicated, adult, male patients with TS “only” (without comorbid psychiatric disorders) and 20 age- and sex-matched control subjects.

**Results:**

Compared to normal controls, TS patients showed a decrease in the fractional anisotropy index (FA) bilaterally in the medial frontal gyrus, the pars opercularis of the left inferior frontal gyrus, the middle occipital gyrus, the right cingulate gyrus, and the medial premotor cortex. Increased apparent diffusion coefficient (ADC) maps were detected in the left cingulate gyrus, prefrontal areas, left precentral gyrus, and left putamen. There was a negative correlation between tic severity and FA values in the left superior frontal gyrus, medial frontal gyrus bilaterally, cingulate gyrus bilaterally, and ventral posterior lateral nucleus of the right thalamus, and a positive correlation in the body of the corpus callosum, left thalamus, right superior temporal gyrus, and left parahippocampal gyrus. There was also a positive correlation between regional ADC values and tic severity in the left cingulate gyrus, putamen bilaterally, medial frontal gyrus bilaterally, left precentral gyrus, and ventral anterior nucleus of the left thalamus.

**Conclusions:**

Our results confirm prior studies suggesting that tics are caused by alterations in prefrontal areas, thalamus and putamen, while changes in the cingulate gyrus seem to reflect secondary compensatory mechanisms. Due to the study design, influences from comorbidities, gender, medication and age can be excluded.

## Background

Gilles de la Tourette syndrome (TS) is a chronic motor and vocal tic disorder of unknown origin. Volumetric MRI studies demonstrated volume changes in brain regions associated to cortico-striato-thalamo-cortical circuits (CSTC) [1], but there is only a limited number of studies available using diffusion tensor imaging (DTI), a sensitive method to detect microstructural changes in white matter. DTI provides information about the directional organization of brain tissue by measuring water diffusion. To characterize the degree of anisotropy and structural organization in the brain the fractional anisotropy (FA) and the apparent diffusion coefficient (ADC) can be used. In most of the DTI studies that have been performed so far in TS, only children have been included. Findings in children are consistent with the hypothesis of dysfunctional CSTC demonstrating reduced FA in the corpus callosum (CC) [2], increased thickness and tract density in the prefrontal region [3], increased parallel and mean diffusivity in the putamen bilaterally [4,5], increased perpendicular diffusivity in the right thalamus [5], reduced anisotropy in the bilateral thalamus [4,5], decreased FA in the left globus pallidus [4], and increased ADC in the bilateral caudate nucleus [4]. Furthermore reduced FA and reduced parallel diffusivity (but increased perpendicular diffusivity) was found in the caudate nucleus bilaterally [6], decreased connectivity between the caudate nucleus and the anterior dorsolateral frontal cortex (when using probalistic fiber tracking) [7] and increased ADC in the corticostriatal pathway (when combining DTI with tract-based spatial statistics (TBSS)) [8].

In addition, there are six studies available using DTI to investigate *adult* TS patients compared to normal controls. It can be assumed that results in children cannot be compared directly to data obtained in adult patients: On the one hand, adults with *persistent* tics probably constitute a subgroup of patients with TS; on the other hand, microstructural alterations that can be detected by DTI – as well as functional changes identified by BOLD functional MRI - in adults are likely to be caused not only by the underlying pathophysiology, but also by secondary compensatory neuroplastic processes. Cavanna et al. [9] found reduced FA in the CC in an adult monocygotic twin with TS “only” (without comorbidities) compared to his unaffected co-twin. Bäumer et al. [10] used a combined approach with transcranial magnetic stimulation (TMS) and DTI in 14 unmedicated adult patients with TS “only” and found an abnormal interhemispheric connectivity in TS compared to normal controls demonstrating not only weaker left-to-right (than right-to-left) interhemispheric inhibitions in TS patients, but also reduced left-to-right interhemispheric inhibitions in TS compared to normal controls. In a study by Thomalla et al. [11] a bilateral FA increase in the white matter was demonstrated in 15 unmedicated adults with TS “only” in the regions underlying the post- and precentral gyrus, below the left supplementary motor area and in the right thalamus (ventro-postero-lateral part). Using probalistic fiber tracking, in addition, structural alterations in somatosensory pathways could be demonstrated [11]. Neuner et al. [12] investigated the microstructure of gray matter nuclei in 15 adult patients with TS “only” and found no differences in the FA and the diffusion parameters between patients and normal controls in the basal ganglia and thalamus, but a positive correlation between diffusion indices and tic severity in the left nucleus accumbens, the right amygdale, the globus pallidus bilaterally, and the left putamen. In another study, the same group found decreased FA and increased radial diffusivity in the corticospinal tract, the CC and long association fibre tracts when using TBSS in 19 adults with TS [13]. Using cortical thickness estimation and voxel-based analysis of T1- and diffusion-weighted structural MRI, Draganski et al. [14] examined 40 adults with TS and found grey matter volume/cortical thickness decrease in prefrontal and limbic structures bilaterally. Cortical thinning extended into the limbic mesial temporal lobe. Increase in somatosensory cortex depended on the intensity of premonitory urges, while decrease in prefrontal cortical thickness correlated negatively with tic severity. In addition, white matter abnormalities were detected including changes in fibre integrity within the CC and in subcortical tracts corresponding to frontal and parietal portions of superior longitudinal fascicle.

Taking available results together obtained from DTI neuroimaging studies in TS patients, it can be concluded that in both children and adults microstructural changes can be detected in the CC, different parts of the basal ganglia, thalamus, frontal regions, and the connectivity in cortico (fronto)-striatal pathways. In adults, in addition, FA and ADC were altered in the limbic system, somatosensory pathways, and the corticospinal tract. Thus, from recent studies it is suggested that these latter brain regions are involved in compensatory processes related to age-dependent tic reduction and voluntary tic suppression.

This study was initiated to further investigate microstructural changes using DTI in adult, unmedicated patients with TS “only”. In a recent volumetric MRI study using magnetization transfer imaging (MTI) and voxel-based morphometry (VBM) in the same group of patients, we were able to detect abnormalities predominantly in the prefrontal and anterior cingulate cortex, and to a lesser degree in the caudate nucleus and the CC [15]. Using DTI, our hypothesis was to detect alterations in the same brain regions.

## Results

### Group comparisons

#### Fractional anisotropy (FA)

**Figure 1:**
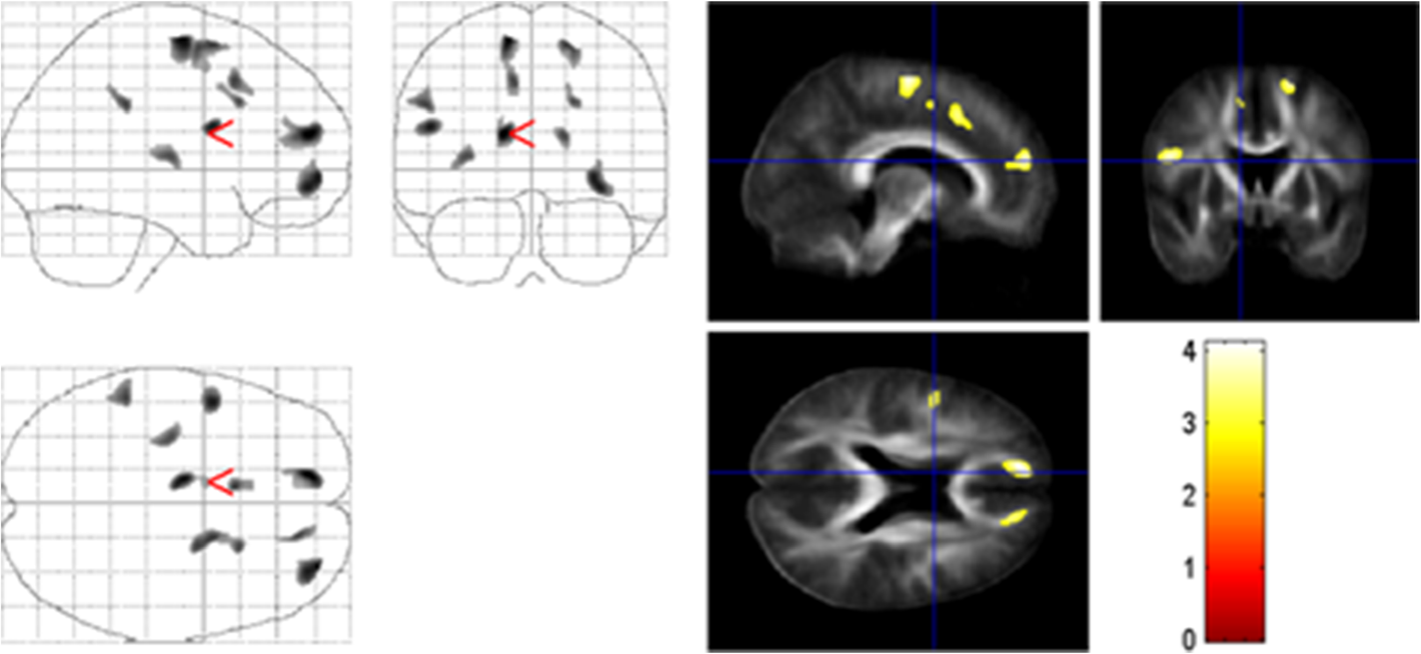
**Group comparison of TS and normal controls.** FA results are superimposed on the mean of all subjects of spatially normalized anisotropy images. Voxels with a significant decrease in FA in TS patients versus controls were found in the white matter of the medial prefrontal cortex below BA 10, the left inferior frontal gyrus (below BA 44), and the cingulate gyrus (p < 0.001, uncorrected).

Compared to normal controls, patients with TS showed significantly reduced regional FA in the white matter of the frontal, parietal, occipital, and limbic lobe. In particular, FA was reduced bilaterally in the medial frontal gyrus, the pars opercularis of the left inferior frontal gyrus (below BA 44), the middle occipital gyrus, the right cingulate gyrus, and the medial premotor cortex underlying BA 6 (p < 0.05, each) (Figure 1, Table 1). No regions displayed significant regional increases in FA in TS patients compared to control subjects.

**Table 1:** Differences in FA and ADC in patients with TS compared to controls

**Anatomical location**	**Cluster**	**MNI-space**			***t*-value**	***p*-value**
	**(voxel)**	***x***	***y***	***z***		**(SVC)**
**FA TS < controls**						
R middle occipital gyrus	112	44	−77	−1	4.20	0.010
L medial frontal gyrus	355	−14	52	18	4.10	0.012
L medial frontal gyrus	238	−11	−10	64	3.94	0.017
L inferior frontal gyrus	172	−50	4	22	3.87	0.021
R superior frontal gyrus	379	31	56	−4	3.87	0.021
R superior frontal gyrus	110	17	4	62	3.66	0.033
L cingulate gyrus	45	−8	15	44	3.59	0.038
L inferior parietal lobe	50	−52	−39	32	3.59	0.038
R medial frontal gyrus	41	16	47	17	3.55	0.041
R cingulate gyrus	15	21	18	34	3.50	0.046
L claustrum	60	−33	−16	7	3.50	0.046
**ADC TS > controls**						
L precentral gyrus	458	−50	13	6	4.86	0.002
L cingulate gyrus	597	−6	−19	30	4.53	0.004
L inferior frontal gyrus	264	−47	4	23	4.27	0.008
L claustrum	171	−34	−2	−6	4.06	0.013
R middle frontal gyrus	71	31	51	−9	3.69	0.029
R medial frontal gyrus	100	13	52	21	3.60	0.035
L postcentral gyrus	20	−53	−16	53	3.55	0.039
R middle frontal gyrus	26	40	42	−10	3.50	0.043

#### Apparent diffusion coefficient (ADC)

**Figure 2:**
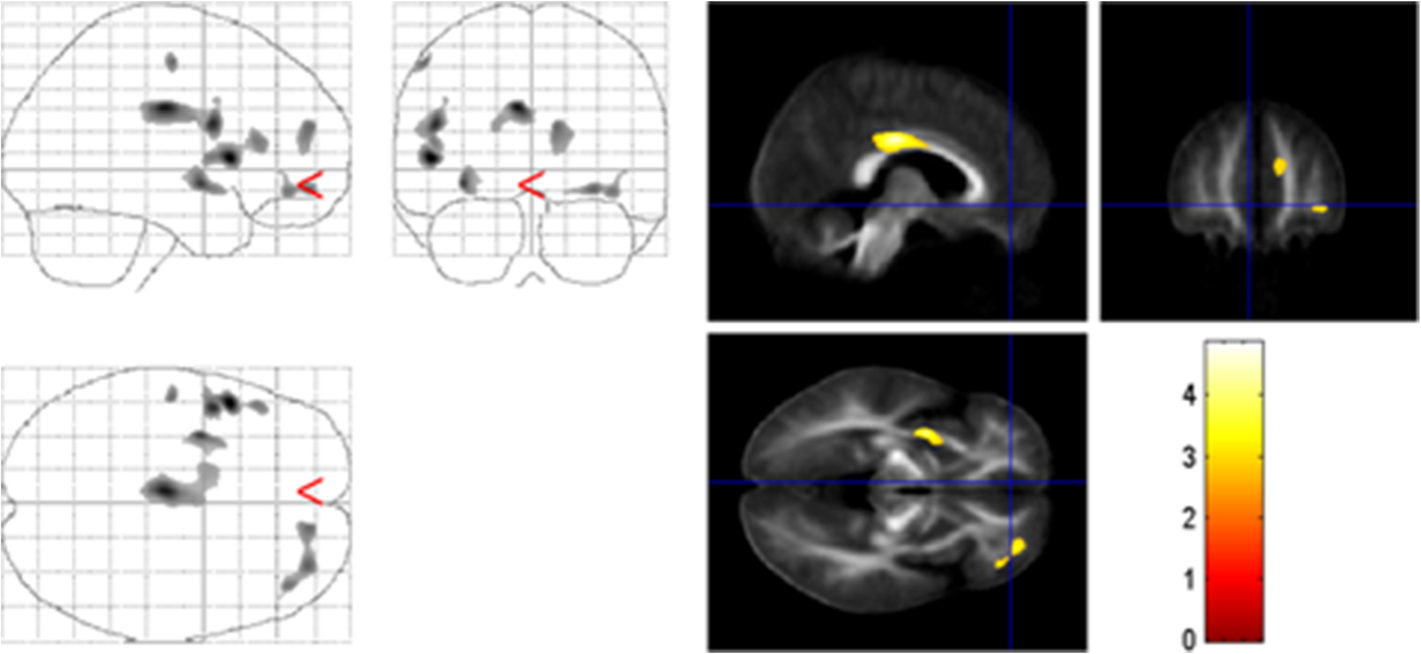
**Group comparison of TS and normal controls.** ADC results are superimposed on the mean of all subjects of spatially normalized anisotropy images. Voxels with a significant increase in ADC in TS patients versus controls were found in the white matter of the left inferior frontal and right medial frontal gyrus, the left cingulate gyrus, the left precentral gyrus, and the claustrum (p < 0.001, uncorrected).

Patients displayed increased mean diffusivity in the white matter of the frontal and parietal lobe. The ADC was significantly increased in the left cingulate gyrus (p < 0.01), prefrontal areas corresponding to BA 10 and 11 (p < 0.01 and 0.05, respectively), and the left precentral gyrus below BA 44 (p < 0.01). In addition, there was a cluster of increased diffusivity in the left putamen with a local maximum in the claustrum (p < 0.05). No significant decreases in ADC maps were found in TS patients compared to controls (Figure 2, Table 1).

Thus, both FA decrease and ADC increase could be detected in the right medial frontal gyrus, the left inferior frontal gyrus, the left cingulate gyrus and the left claustrum.

### Correlations between FA and ADC maps with tic severity

**Figure 3:**
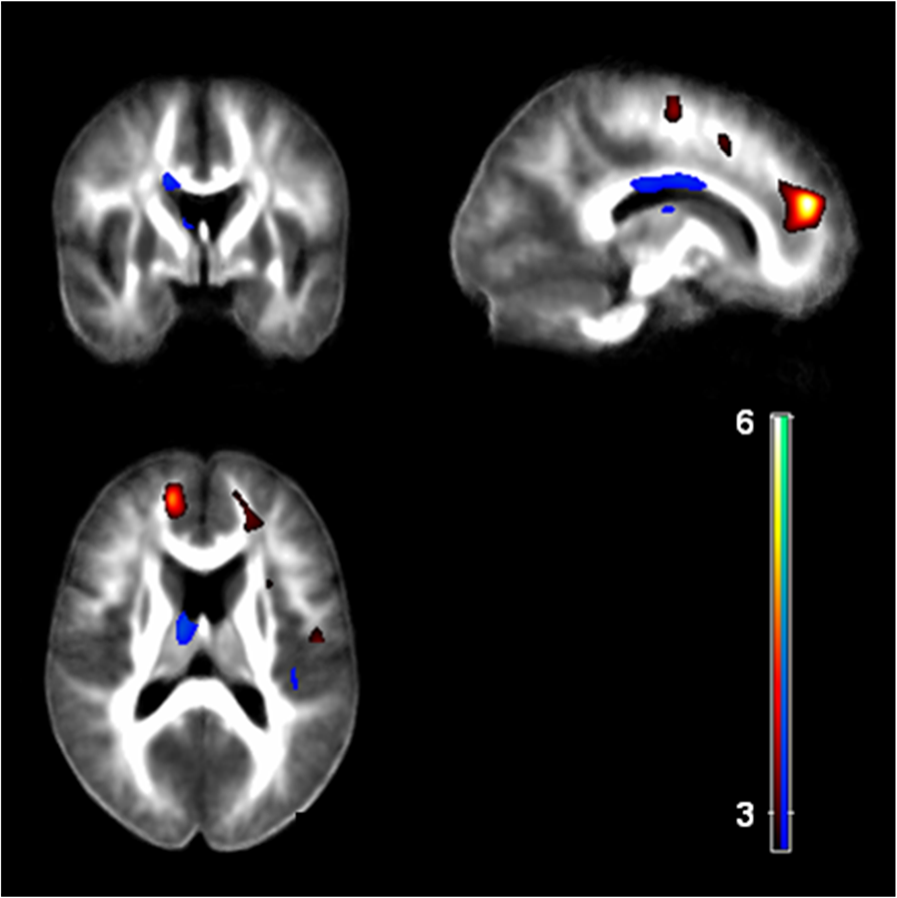
**Correlation of FA with tic severity.** There was a negative correlation (red) between tic severity (according to YGTSS) and the FA in prefrontal areas, the cingulate gyrus, and the right thalamus. Tic severity correlated positively with FA (blue) in the body of the CC, the left thalamus, and the right superior temporal gyrus (p < 0.001, uncorrected).

Tic severity (as assessed by YGTSS) was negatively correlated with FA values in the frontal, occipital and limbic lobe: the left superior frontal gyrus (below BA 10), the medial frontal gyrus bilaterally (below BA 6), the cingulate gyrus bilaterally, and the ventral posterior lateral nucleus of the right thalamus. A positive correlation between tic severity and FA was found in the body of the CC, the left thalamus, the right superior temporal gyrus and the left parahippocampal gyrus (Figure 3, Table 2).

**Table 2:** Brain regions showing significant correlations with tic severity

**Anatomical location**	**Cluster**	**MNI-space**			**t-value**	**p-value**
	**(voxel)**	***x***	***y***	***z***		**(SVC)**
**FA negative correlation**						
L superior frontal gyrus	1394	−15	51	19	6.29	0.008
L cingulate gyrus	291	−10	16	46	4.19	0.010
R middle occipital gyrus	119	44	−76	−2	3.81	0.024
R middle frontal gyrus	186	28	43	4	3.69	0.031
R thalamus (VPL)	71	20	−21	−4	3.65	0.033
R cingulate gyrus	28	24	38	16	3.52	0.044
L medial frontal gyrus	22	−13	−8	61	3.48	0.045
R medial frontal gyrus	12	9	−25	67	3.46	0.051
**FA positive correlation**						
R superior temporal gyrus	92	54	−7	−2	4.36	0.001
L lingual gyrus	627	−19	−67	−11	4.04	0.014
L parahippocampal gyrus		−29	−53	−4	3.96	0.017
R cingulate gyrus	75	5	22	32	3.92	0.019
R superior temporal gyrus	345	43	−23	9	3.78	0.025
L postcentral gyrus	123	−41	−23	47	3.69	0.031
L corpus callosum (body)	306	−17	−6	31	3.67	0.032
L thalamus	131	−11	−9	13	3.66	0.033
R medial frontal gyrus	25	7	40	−11	3.61	0.037
**ADC positive correlation**						
L Putamen	2185	−26	4	−4	4.39	0.006
R Putamen	1192	28	2	−5	4.29	0.007
L precentral gyrus	297	−52	7	10	3.85	0.021
R putamen	190	21	18	4	3.75	0.026
L middle occipital gyrus	143	−50	−58	−11	3.71	0.026
L anterior cingulate gyrus	49	−9	44	6	3.65	0.031
L caudate body	109	−19	4	20	3.59	0.036
R precentral gyrus	27	43	3	10	3.57	0.038
L cingulate gyrus	51	−7	−20	29	3.56	0.038
R medial frontal gyrus	56	11	50	5	3.54	0.040
R middle frontal gyrus	19	29	50	−10	3.48	0.045
L Thalamus (VA)	17	−9	−2	9	3.42	0.051

**Figure 4:**
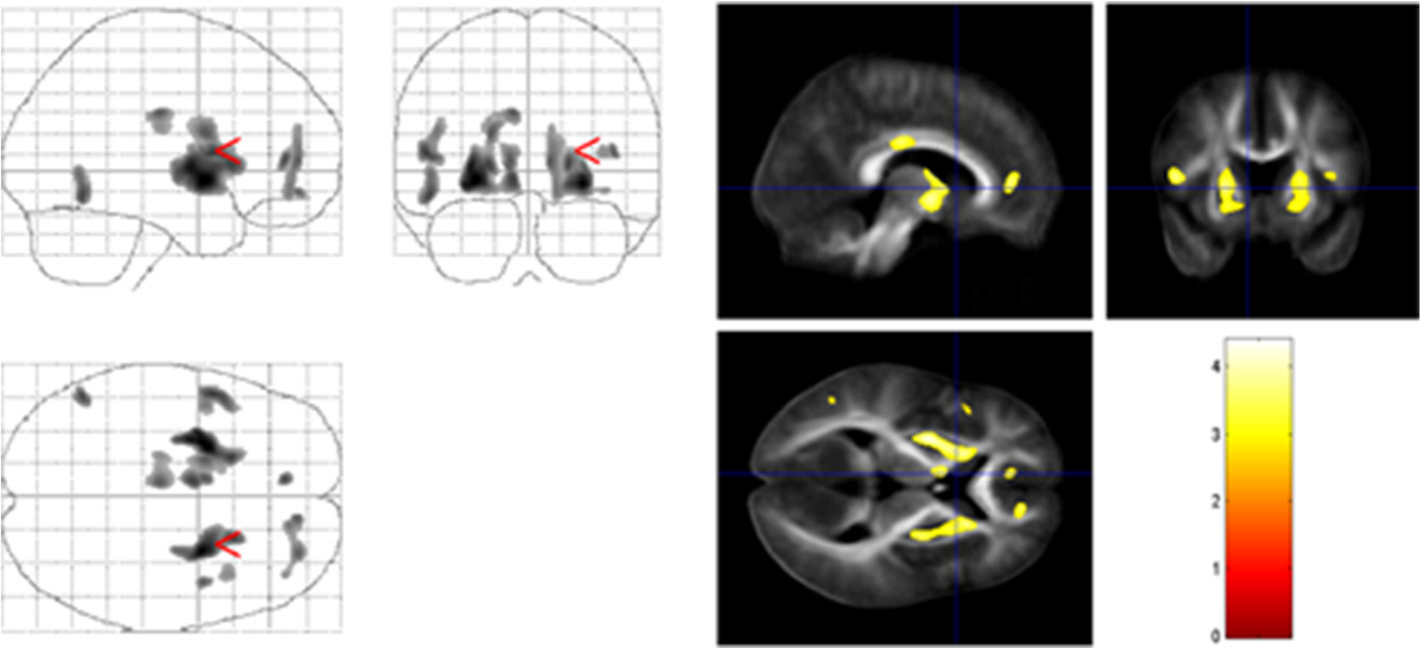
**Correlation of ADC with tic severity.** Tic severity (according to YGTSS) and the ADC correlated positively in the putamen bilaterally, the medial frontal gyrus bilaterally, and the left thalamus (p < 0.001, uncorrected).

When correlating regional ADC values and tic severity (YGTTS), we found a positive correlation in the left cingulate gyrus, the putamen bilaterally, the medial frontal gyrus bilaterally, the left precentral gyrus (BA 44) as well as the ventral anterior nucleus of the left thalamus (Figure 4, Table 2).

## Discussion

Using DTI, we found microstructural changes in the white matter of prefrontal areas, the pars opercularis of the left inferior frontal gyrus, the cingulate gyrus, the medial premotor cortex, the left precentral gyrus, and the left putamen. For the following brain regions there was a positive correlation between tic severity and FA and ADC values, respectively: body of the CC, left thalamus, right superior temporal gyrus, putamen bilaterally, left parahippocampal gyrus, left cingulate gyrus, medial frontal gyrus bilaterally, and left precentral gyrus (Figure 4, Table 2). In addition, we found a negative correlation between FA values and tic severity in the left superior frontal gyrus, medial frontal gyrus bilaterally, cingulate gyrus bilaterally, and the ventral posterior lateral nucleus of the right thalamus. Thus, most of those brain regions that showed abnormalities in structural organization, also demonstrated a correlation with tic severity. Furthermore, these results obtained by DTI are in broad agreement with our prior findings in the same group of patients using VBM and MTI [15]. In addition, most of these alterations have been described before in other DTI studies investigating adults with TS including the CC [9,10,13], precentral gyrus [11], thalamus [11,12], left putamen [12], and limbic regions [12].

Comparable to our recent VBM/MTI study in the same group of unmedicated adult patients with TS “only”, we detected the most prominent changes in different frontal areas, particularly in medial and inferior prefrontal areas (reduced regional FA and increased mean diffusivity) including the left pars opercularis (reduced regional FA) and the left precentral gyrus (increased mean diffusivity). These results are in line with several other studies using both functional and structural neuroimaging techniques [1,16]. Since our group of patients comprised only patients with TS “only” without comorbidities, alterations in these frontal regions in TS patients have to be related to the presence of tics (and not exclusively to the presence of comorbidities such as attention deficit hyperactivity disorder (ADHD) and obsessive compulsive disorder (OCD)). This assumption is corroborated by the fact that we found not only reduced regional FA and increased mean diffusivity in medial and inferior prefrontal areas, respectively, but also a positive correlation between tic severity and regional ADC values in the medial frontal gyrus bilaterally and the left precentral gyrus. Since, we detected, in addition, a negative correlation between FA values and tic severity in prefrontal areas bilaterally, it can be hypothesized that - at least in adult patients - these frontal regions are involved not only in tic generation but also in tic suppression or compensatory adaptation mechanisms resulting in tic decline with increasing age as suggested earlier [1].

Our data further corroborate an involvement of the CC in the generation of tics in adult TS patients as suggested earlier: in both children and adults, larger CC volumes [17-19] and reduced FA [2,9,13,14] have been reported as well as an altered structure-function relationship in the motor CC in adults with TS “only” using a combined TMS-DTI approach [10]. Since we found a positive correlation between tic severity and FA values in the CC, it can be speculated that CC alterations are correlated to neurodevelopmental abnormalities - resulting in reduced transcallosal inhibition of cortical neurons - rather than adaptive mechanisms to compensate for impairments in other brain regions [9]. This interpretation is further supported by the fact that even in children a negative correlation has been reported between tic severity and CC volumes [19,20].

Comparable to our recent study in the same group of patients [15], we detected abnormalities in the cingulate gyrus. Using VBM and MTI we found reduced gray and white matter cingulate volumes [15]; in this study using DTI reduced regional FA and increased mean diffusivity were obvious. We hypothesize that these changes represent secondary compensatory mechanisms, because (1) in both of our studies using three different MRI techniques we found a negative correlation between tic severity and changes in the cingulate gyrus, (2) in functional studies it could be demonstrated that the cingulate is activated during tic inhibition [21-23], (3) the cingulate gyrus has numerous interconnections with the prefrontal and orbitofrontal cortex, motor systems, and the striatum – regions that have been suggested to be involved in tic generation [1], and (4) there is evidence for an involvement of the cingulate gyrus in the initiation and motivation of goal-directed behaviours [24]. Furthermore, DTI studies in children failed to detect abnormalities in the cingulate gyrus.

In accordance with other DTI studies in adult TS patients, in addition, we found alteration in the thalamus [11,12] and the putamen [12]. These brain regions have been extensively described in association with TS pathophysiology in adults and children not only when using DTI [4,5], but also when using other MRI techniques such as VBM [17,25]. Accordingly, the thalamus is the most often used target for deep brain stimulation in adult patients suffering from severe treatment resistant TS [26]. Although some studies in both children with TS “only” (using VBM) [27] and neuroleptic-naïve adults with TS or chronic motor tics with and without comorbidities (using large-deformation high dimensional brain mapping) [28] failed to detect changes in the basal ganglia and thalamus, today there is little doubt that these brain regions are involved in the pathophysiology of tics.

As described before by Thomalla et al. [11] in a group of 15 unmedicated adults with TS “only” and Draganski et al. [14] (in a mixed group of TS patients with and without comorbidities and medication), we were able to detect altered FA in the precentral gyrus, a brain region that can be assigned to networks involved in sensory-motor processing [11]. However, in contrast to our results and those from other studies [4,13], Thomalla et al. [11] and Draganski et al. [14] found an *increased* (and not reduced) FA with ADC decrease in the somatosensory cortex region bilaterally and in other brain areas such as the thalamus [11]. These conflicting results could either be related to methodological differences in the different cross-sectional studies or be caused by different groups of patients (e.g. with respect to disease duration). This problem can only be solved by follow-up studies investigating whether the FA changes over time and may reflect compensatory structural changes in patients with TS. In addition, until today it is unclear which histological changes correlate to these alterations detected by DTI. The involvement of the precentral gyrus in the pathophysiology of tics is further supported by the fact that we found not only a reduced FA, but also a positive correlation between the ADC value in the left precentral gyrus (BA 44) and the tic severity.

Our finding of a reduced regional FA in the left pars opercularis should receive special attention. The inferior frontal gyrus consists of three distinct subparts: the pars opercularis, the pars triangularis, and the pars orbicularis. There is substantial evidence that the pars opercularis is not only involved in language and music (for review see [29]), but also in motor processing. In particular, the pars opercularis seems to be a key component in the human mirror neuron system that is activated during action observation and imitation [30]. In accordance with this assumption, a significant volume reduction of the pars opercularis could be demonstrated in patients suffering from high-functioning autism spectrum disorders suggesting that this brain region also plays an important role in social (dys-)function [31]. In addition, the role of the pars opercularis in inhibitory control has been demonstrated using repetitive transcranial magnetic stimulation (rTMS) [32]. In accordance with these results, structural abnormalities in the pars opercularis have been demonstrated in children suffering from ADHD suggesting that developmental abnormalities of the pars opercularis lead to inhibition difficulties [33]. In TS, therefore, it is conceivable that the pars opercularis is involved in inhibitory control mechanisms and in particular in the generation of complex motor and vocal tics such as copro- and echophenomena. Accordingly, it has been suggested that echophenomena might be caused by a dysfunction of the mirror neuron system [34,35].

It should be mentioned that studies using newer techniques (for example TBSS, cortical thickness measurement, probalistic fiber tracking) resulted in additional findings demonstrating alterations in somatosensory pathways [11,14], long association fibre tracts such as the inferior fronto-occipitalis fascicle, the superior longitudinal fascicle and the fascicle uncinatus [13] and cortical thinning in prefrontal and limbic structures [14], respectively.

The following limitations of the study have to be addressed: Firstly, we investigated a carefully selected group of patients including only adult, unmedicated male with TS “only” without comorbidities. Since influences from sex, age, medication, and comorbidities can be excluded, it can be concluded that microstructural abnormalities detected in this study are indeed related to the tic disorder. Since the majority of adult patients with TS, however, suffer not only from tics, but also from one or more psychiatric comorbidities [36], it has to remain open whether our group of patients is representative for adults with TS. It is still matter of discussion whether TS is a unitary or heterogeneous condition encompassing different phenotypes (with and without ADHD, aggressive behaviours, OCD, and coprophenomena) [37]. Secondly, comparable to most other neuroimaging studies in TS, our sample size was relatively small. However, using DTI a sample size of at least 15 subjects is regarded as suitable for reliable analyses. In all but one DTI study performed so far in adults with TS a smaller number of patients has been included. Thirdly, we demonstrated uncorrected data. However, our results were supported by recent findings in the same group of patients using both VBM and MTI [15]. Fourthly, in this study SPM2 was used to analyze the data. One might argue that a better segmentation and registration tool might provide more reliable findings. To address the issue of compensatory mechanisms in TS in more detail a prospective follow-up study is necessary.

## Conclusion

In this study we used DTI to investigate microstructural integrity of white matter pathways and brain tissue in a relatively large group of unmedicated, adult patients with TS “only”. Our results are in line with recent findings in a small number of studies suggesting that tics are caused by alterations in prefrontal areas, thalamus and putamen. It can be hypothesized that additional changes in the cingulate gyrus are related to secondary compensatory mechanisms.

## Methods

### Patients and normal controls

19 unmedicated right handed male patients with TS (mean age = 30.4, range 18–60, median = 27, SD = 11.0) and 20 control subjects matched for age, sex and handedness (mean age = 31.7, range 18–65, median = 27, SD = 10.9; p = 0.71) were included in the study. In all patients a physical and neurological examination, a psychiatric interview, and a routine blood laboratory tests were done by one of the investigators (KMV) to exclude other neurological or psychiatric disorders. All patients fulfilled diagnostic criteria for TS according to DSM-IV-TR criteria. 12 patients were drug-naïve; all other patients were drug free for a least 1 year (only one patient for 5 months) before entering the study. Using the Yale Gobal Tic Severity Scale (YGTSS) [38] mean disease severity was 28.8 (range, 9–69). None of the patients fulfilled diagnostic criteria for one of the following co-morbid disorders: depression, anxiety, addiction, self-injurious behavior, OCD, and ADHD. For diagnosing OCD we used a clinical interview in combination with the German version of the Yale-Brown obsessive compulsive scale (Y-BOCS) [39]. Using the Y-BOCS, we found no or subclinical OCD in 18 patients and mild OCD in one patient. Although the short version of the German version of the Wender Utah rating scale (WURS-k) [40] suggested the diagnosis of ADHD in 4 patients, none of them fulfilled diagnostic criteria for ADHD according to DSM IV-TR criteria (neither at present time nor in childhood).

This study was approved by the local ethic committees of the Hannover Medical School and the Otto-von-Guericke-University Magdeburg, Germany. After complete description of the study to the subjects, written informed consent was obtained.

### MRI acquisition protocol

Images were acquired on a neuro-optimized 1.5-T GE Signa Horizon LX (General Electric Company, Milwaukee, WI, USA) using a 3-dimensional T1-weighted spoiled gradient recalled echo (SPGR) sequence. A standard quadratur birdcage GE head coil was used for both the radio-frequency transmission and nuclear magnetic resonance signal reception. DTI was performed using spin-echo based echoplanar imaging (EPI) covering the whole brain (TR 10 s; TE 70 ms; 128 × 128 acquisition matrix, interpolated by zeropadding to 256 × 256, FOV 28 cm, 39 axial slices, without interslice gap, 3 mm thickness). The data for diffusion tensor calculations were collected with 12 noncollinear gradient orientations (b = 1000 s/mm^2^, each additionally measured with the opposite diffusion gradient polarity). Diffusion-weighting was realized by the standard PGSE method with two trapezoidal diffusion gradients of 32.2 ms duration and 38.4 ms separation of their leading edges. The default double RF pulses of the Signa scanner were used. Twelve noncollinear gradient orientations were chosen according to the DTI acquisition scheme proposed by Papadakis et al. [41] and the values specified by Skare et al. [42] using a single b-value of 1000 s/mm2. Again, each of the 12 directions was additionally recorded with the opposite diffusion gradient polarity. To reduce the probability of head motion between each of the two opposite gradient measurements, they were performed consecutively. A total of 24 diffusion-weighted measurements, four averages each, were divided into four blocks, each preceded by a non-diffusion-weighted acquisition. The total scan time was 16 min [41]. During scanning, all participants were comfortably placed and their heads were fixated within the headcoil with special cushions. All subjects received additional T2-weighted images to exclude cerebrovascular disease, which was normal in all subjects studied according to standard clinical neuroradiological criteria on visual inspection.

### Image processing

On a Sun workstation the DTI images were eddy-current-corrected according to the correction scheme by Bodammer et al. [43] followed by a correction for head motion based on the non-diffusion-weighted images using the AIR software package [44]. Diffusion tensors were calculated for each voxel by singular value decomposition and then decomposed into eigenvalues and eigenvectors. The self written algorithm was coded in MATLAB. On the basis of the eigenvalues ADC and FA maps were computed. After that data were processed on a standard IBM-compatible PC using SPM2 statistical parametric mapping software (Welcome Department of Cognitive Neurology, London) and working in an analysis environment (MATLAB version 6.1; the Math Works Inc, Natick, Mass). The images were reoriented into oblique axial slices aligned parallel to the anterior-posterior commissural axis with the origin set to the anterior commissure. The FA and ADC maps were pre-processed and analyzed by SPM2 using an approach adopted from VBM introduced by Good and colleagues [45]. This included an optimized normalization procedure (to MNI space), automated removal of skull and CSF signals as well as smoothing (8-mm FWHM with Gaussian filter) [46].

All non-diffusion-weighted EPI scans were normalized to the mean T2-weighted EPI-template provided by SPM and further used to create a site-specific EPI template appropriate to the population sample and with scanner specific image contrast. This site-specific template was again used for normalization and brain extraction for the individual images in the group studied. This resulted in optimally normalized FA and ADC maps removed from non-brain structures. Images were smoothed to 10 mm using a full-width-half-maximum Gaussian filter.

### Statistical analysis

Processed images of FA and ADC were analyzed in the framework of the general linear model. This framework allows the testing, on a voxel-by-voxel basis, of the null hypothesis that the FA and ADC maps in the two populations (patients and controls) are the same. The resulting statistical parameters constitute a SPM of the t statistic (SPM (t)). Group comparison of TS patients and healthy controls was performed in SPM2 using the model ‘compare-populations: one scan/subject (ANCOVA)’ with the global mean voxel value as confounding factor. Additionally, regression analyses with clinical measures were explored using the SPM2 model ‘single subject: covariates only’.

Resulting statistical parametric maps of FA and ADC were derived at a significance level of p < 0.001, uncorrected with an extent threshold of 10 continuous voxels.

For regions where an effect was hypothesized, namely the fronto-striatal and limbic system (see introduction), a small volume correction (SVC) limited to the volume of that particular region was performed [47,48]. Here, we controlled for multiple comparisons by using the family wise error (FWE) method (p < 0.05).

### Availability of supporting data

The data set supporting the results of this article is included within the article.

## Competing interests

The authors declare that they have no competing interests.

## Authors’ contributions

KMV participated in the design of the study, investigated and rated the patients, and drafted the manuscript. TP participated in the design of the study, carried out the MR imaging, performed the statistical analysis, and helped to draft the manuscript. JG participated in the design of the study and helped to perform the statistical analysis and to draft the manuscript. NB carried out the MR imaging and helped to perform the statistical analysis and to draft the manuscript. JK carried out the MR imaging and helped to perform the statistical analysis and to draft the manuscript. TPrell helped to perform the statistical analysis and to draft the manuscript. All authors read and approved the final manuscript.
